# On multipliers of matrix domains

**DOI:** 10.1186/s13660-018-1887-4

**Published:** 2018-10-29

**Authors:** Gholamreza Talebi

**Affiliations:** Department of Mathematics, Vali-e-Asr University of Rafsanjan, Rafsanjan, Islamic Republic of Iran

**Keywords:** 46A20, 46A45, 40H05, Köthe–Toeplitz duals, Summability matrix, Sequence space

## Abstract

In this paper, we obtain the Köthe–Toeplitz duals of the domain of an arbitrary invertible summability matrix *E* in the space $\ell_{p}$. As a consequence, we apply our results to the Fibonacci and Euler sequence spaces and show that some recent works by Altay, Başar, and Mursaleen (Inf. Sci. 176:1450–1462, 2006) are all the special cases of our results.

## Introduction and preliminaries

Let *ω*, $\ell_{\infty}$, $\ell_{p}$, and *c* be the sets of all sequences, bounded sequences, *p*-absolutely summable sequences, and convergent sequences, respectively. The multiplier space of the sequence spaces *X* and *Y* is defined by

1.1 $$\begin{aligned} \mathfrak{M} ( {X ,Y } ) = \bigl\{ {z = ( {{z_{n}}} )\in \omega: xz = ( {{x_{n}} {z_{n}}} ) \in Y , \forall x = ( {{x_{n}}} ) \in X } \bigr\} , \end{aligned}$$

and the *α*-, *β*-, and *γ*-duals of the space *X*, which are denoted by $X^{\alpha}$, $X^{\beta}$, and $X^{\gamma}$, are

$${X ^{\alpha}} :=\mathfrak{M} ( {X ,{\ell_{1}}} ),\qquad {X ^{\beta}}: = \mathfrak{M} ( {X ,cs} ),\quad \mbox{and}\quad {X^{\gamma}}: = \mathfrak{M} ( {X ,bs} ). $$

Here

$$bs = \Biggl\{ { ( {{x_{n}}} ) \in\omega: \Vert x \Vert _{bs} = \sup_{n} \Biggl\vert {\sum _{k = 0}^{n} {{x_{k}}} } \Biggr\vert < \infty} \Biggr\} $$

and

$$cs = \Biggl\{ { ( {{x_{n}}} ) \in\omega: \Biggl( {\sum _{k = 0}^{n} {{x_{k}}} } \Biggr)_{n} \in c} \Biggr\} . $$

For an infinite matrix *A*, the domain of *A* in the space *X*, which is a sequence space, is defined by

$$\begin{aligned} {X_{A}} = \{ {x \in\omega, Ax \in X} \}. \end{aligned}$$

Recently in [9], the author defined and studied the domain of an arbitrary invertible summability matrix $E=(E_{n,k})_{n,k\geq0}$ in the space $\ell_{p}$, i.e., $E_{p}:=(\ell_{p})_{E}$. One can easily show that the sequence space $E_{p}$ is a normed space with ${ \Vert x \Vert _{{E_{p}}}}: = { \Vert {{E}x} \Vert _{{\ell_{p}}}}$, and the inclusion $E_{q}\subseteq E_{p}$ holds while $1\le q\le p$. Moreover, applying Hölder’s inequality, we have

$$\begin{aligned} { \Vert x \Vert _{{E_{p}}}} \le \Bigl( {\sup_{k \in\mathbb{N} } {{ \bigl\Vert {{{ \{ {{E_{n,k}}} \}}_{n \in\mathbb{N}}}} \bigr\Vert }_{{\ell_{1}}}}} \Bigr)^{1/p} { \Vert x \Vert _{{\ell_{p}}}}, \end{aligned}$$

which implies the inclusion $\ell_{p}\subseteq E_{p}$ for $1\le p<\infty$ provided that

$$\sup_{k \in\mathbb{N}} { \bigl\Vert {{{ \{ {{E_{n,k}}} \} }_{n \in{\mathbb{N}} }}} \bigr\Vert _{{\ell_{1}}}} < \infty. $$

Eventually, one can easily check that if the map $E: E_{p}\to\ell_{p}$ is onto, then the space $E_{p}$ is linearly isomorphic to $\ell_{p}$ and in such a case the columns of the matrix $E^{-1}$ form a Schauder basis for $E_{p}$, where $1\le p<\infty$.

It is known that, for the infinite summability matrix *E*, there may be left or right inverses, or even if both exist, they may not be unique. In this paper we deal with the case in which the left and right inverses are equal, and we denote it by $E^{-1}$. Further, to give full knowledge on the definitions and calculations with infinite matrices, we refer the readers to the textbook [3].

In this paper, we are going to find out the *α*-, *β*-, and *γ*-duals of the space $E_{p}$ for $p\in[1,\infty]$. We assume throughout that $\mathfrak{F}$ is the collection of all finite subsets of $\mathbb{N}$ and $\frac{1}{p} + \frac{1}{q} = 1$. Further, we denote by $(X:Y)$ the class of all infinite matrices which transform *X* into *Y*.

## Main results

In this section, we assume that the transformation $E: E_{p}\to\ell_{p}$ is surjective and state theorems determining the *α*-, *β*-, and *γ*-duals of $E_{p}$, where $p\in[1,\infty]$. We consider only the case $1 < p < \infty$ in the proof of Theorems 2.1–2.3 below, because the cases $p = 1$ and $p =\infty $ can be proved similarly.

### Theorem 2.1

*Define the sets* $G_{q}$ *and* $G_{\infty}$ *as follows*:

$$G_{q}= \biggl\{ { ( {{b_{n}}} )\in\omega:\sup _{K \in \mathfrak{F} } \sum_{k} {{{ \biggl\vert { \sum_{n \in K} E_{n,k}^{-1}b_{n} } \biggr\vert }^{q}} < \infty} } \biggr\} $$

*and*

$${G_{\infty}} = \biggl\{ { ( {{b_{n}}} ) \in\omega: \sup _{K \in\mathfrak{F}} \sum_{k} { \biggl\vert { \sum_{n \in K} {E_{n,k}^{ - 1}{b_{n}}} } \biggr\vert < \infty} } \biggr\} . $$

*Then* $(E_{1})^{\alpha}=G_{\infty}$ *and* $(E_{p})^{\alpha}=G_{q}$, *where* $1< p\le \infty$.

### Proof

First, consider the following equations:

2.1 $$\begin{aligned} {b_{n}} {x_{n}} = \sum _{k =0}^{\infty}E_{n,k}^{-1}{b_{n}} {y_{k}} = { ( {{A}y} )_{n}}\quad ( {n \in{\Bbb {N}}} ), \end{aligned}$$

in which the rows of the matrix *A* are the product of the rows of the matrix $E^{-1}$ with the sequence $b=(b_{n})$ and *y* is the *E*-transform of the sequence *x*. Therefore, we realize by (2.1) that $bx=(b_{n}x_{n})\in\ell_{1}$ while $x\in E_{p} $ if and only if $Ay\in\ell_{1}$ whenever $y\in\ell_{p}$. That is, $b=(b_{n})\in (E_{p})^{\alpha}$ if and only if $A\in (\ell_{p}:\ell_{1} )$. So, by 76 of [8], we obtain that

$$\sup_{K \in\mathfrak{F} } \sum_{k} {{{ \biggl\vert {\sum_{n \in K} E_{n,k}^{-1}b_{n} } \biggr\vert }^{q}} < \infty}. $$

This implies that $(E_{p})^{\alpha}=G_{q}$. □

### Theorem 2.2

*Define the sets* $H_{1}$, $H_{2}$, $H_{3}$, *and* $D_{q}$ *by*

$$\begin{aligned} &H_{1} = \Biggl\{ { ( {{b_{n}}} ) \in\omega:\sum _{n = 0}^{\infty}{E_{n,k}^{ - 1}{b_{n}} \textit{ exists for any }} k\in {\mathbb{N} }} \Biggr\} , \\ &{H_{2}} = \Biggl\{ { ( {{b_{n}}} ) \in\omega:\sup _{n,k \in\mathbb{N}} \Biggl\vert {\sum_{j = 0}^{n} {E_{j,k}^{ - 1}{b_{j}}} } \Biggr\vert < \infty} \Biggr\} , \\ &{H_{3}} = \Biggl\{ { ( {{b_{n}}} ) \in\omega:\lim _{n \to \infty} \sum_{k = 0}^{\infty}{ \Biggl\vert {\sum_{j = 0}^{n} {E_{j,k}^{ - 1}{b_{j}}} } \Biggr\vert } = \sum _{k = 0}^{\infty}{ \Biggl\vert {\sum _{j = 0}^{\infty}{E_{j,k}^{ - 1}{b_{j}}} } \Biggr\vert } } \Biggr\} , \end{aligned}$$

*and*

$${D_{q}} = \Biggl\{ { ( {{b_{n}}} ) \in\omega:\sup _{n \in \mathbb{N} } {{\sum_{k = 0}^{n} { \Biggl\vert {\sum_{j = 0}^{n} {E_{j,k}^{ - 1}{b_{j}}} } \Biggr\vert } }^{q}} < \infty} \Biggr\} \quad ( {1 < q < \infty} ). $$

*Then* $(E_{1})^{\beta}=H_{1}\cap H_{2}$, $(E_{\infty})^{\beta}=H_{1}\cap H_{3}$, *and* $(E_{p})^{\beta}=H_{1}\cap D_{q}$, *where* $1< p<\infty$.

### Proof

Consider the equation

2.2 $$\begin{aligned} \begin{aligned}[b] \sum_{k = 0}^{n} {{b_{k}} {x_{k}}} &= \sum_{k = 0}^{n} { \Biggl[ {\sum_{j = 0}^{\infty}E_{k,j}^{-1}y_{j} } \Biggr]} {b_{k}} \\ &= \sum_{k = 0}^{\infty}{ \Biggl[ {\sum _{j = 0}^{n} E_{j,k}^{-1}b_{j} } \Biggr]} {y_{k}}= { ( {{S }y} )_{n}}, \end{aligned} \end{aligned}$$

in which *y* is the *E*-transform of *x* and $S= (s_{n,k} )$ is defined by

2.3 $$\begin{aligned} s_{n,k} = {\sum_{j = 0}^{n} E_{j,k}^{-1}b_{j} } \end{aligned}$$

for all $n,k\in\mathbb{N}$. Accordingly, we derive from (2.2) that $bx=(b_{n}x_{n})\in cs$ whenever $x=(x_{n})\in E_{p}$ if and only if $S y \in c$ while $y=(y_{n})\in\ell_{p}$. This implies that $b=(b_{n})\in (E_{p} )^{\beta}$ if and only if $S\in(\ell _{p}:c)$. Hence, we deduce from 16 of [8] that

$$\begin{aligned} &\sum_{n = 0}^{\infty}E_{n,k}^{ - 1}{b_{n}} \mbox{ exists for any }k \in\mathbb{N} \quad \mbox{and } \\ &\sup_{n \in\mathbb{N} } {{\sum_{k = 0}^{n} { \Biggl\vert {\sum_{j = 0}^{n} {E_{j,k}^{ - 1}{b_{j}}} } \Biggr\vert } }^{q}} < \infty, \end{aligned}$$

which shows that $(E_{p})^{\beta}=H_{1}\cap D_{q}$. □

### Theorem 2.3

*Define the set* $D_{1}$ *by*

$${D_{1}} = \Biggl\{ { ( {{b_{n}}} ) \in\omega :\sup _{n \in \mathbb{N} } {{\sum_{k = 0}^{n} { \Biggl\vert {\sum_{j = 0}^{n} {E_{j,k}^{ - 1}{b_{j}}} } \Biggr\vert } }} < \infty} \Biggr\} . $$

*Then* $(E_{1})^{\gamma}=H_{2}$, $(E_{\infty})^{\gamma}=D_{1}$, *and* $(E_{p})^{\gamma}=D_{q}$, *where* $1< p<\infty$.

### Proof

Using 1, 5, and 6 of [8], the proof can be easily adopted from one of Theorems 2.1 and 2.2 above, and so we omit the details. □

## Special cases

In the following we present several special cases of Theorems 2.1–2.3. First, consider the Fibonacci sequence spaces defined by

$${F_{p}} = \Biggl\{ { ( {{x_{n}}} )\in\omega: \sum _{n = 0}^{\infty}{{{ \Biggl\vert {\frac{1}{{{f_{n}}{f_{n + 1}}}}\sum _{k = 0}^{n} {f_{k}^{2}{x_{k}}} } \Biggr\vert }^{p}}} < \infty} \Biggr\} \quad (1\le p < \infty) $$

and

$${F_{\infty}} = \Biggl\{ { ( {{x_{n}}} )\in\omega: \sup _{n \in\mathbb{N}} {{{ \Biggl\vert {\frac{1}{{{f_{n}}{f_{n + 1}}}}\sum _{k = 0}^{n} {f_{k}^{2}{x_{k}}} } \Biggr\vert }^{p}}} < \infty} \Biggr\} , $$

which are the matrix domain of the Fibonacci matrix in $\ell_{p}$ [5], where the Fibonacci matrix $F=(F_{n,k})_{n,k\ge0}$ is defined by

$$F_{n,k} = \textstyle\begin{cases} \frac{{f_{k}^{2}}}{{{f_{n}}{f_{n + 1}}}},& 0 \le k \le n,\\ 0,& \mbox{otherwise.} \end{cases} $$

Here $\{f_{k}\}_{k=0}^{\infty}$ is a sequence of Fibonacci numbers defined by ${f_{n}} = {f_{n - 1}} + {f_{n - 2}}$ for all $n \ge1$, where $f_{-1}=0$ and $f_{0}=1$. The inverse of the Fibonacci matrix, $F^{-1}=(c_{n,k})$, is

$$c_{n,k}= \textstyle\begin{cases} { ( { - 1} )^{n - k}}\frac{{{f_{k}}{f_{k + 1}}}}{{f_{n}^{2}}},&{k \le n \le k + 1},\\ 0,&\mbox{otherwise.} \end{cases} $$

Applying Theorems 2.1, 2.2, and 2.3, we have the following results.

### Corollary 3.1

*The* *α*-, *β*-, *and* *γ*-*duals of Fibonacci sequence spaces* $F_{p}$ ($1\le p\le\infty$) *are as follows*:

1. ${({F_{1}})^{\alpha}} = \{ { ( {{b_{n}}} ) \in\omega: \sup_{n,k \in{\Bbb {N}}} \sum_{n = k}^{k + 1} { \vert {{{( - 1)}^{n - k}}\frac{{{f_{k}}{f_{k + 1}}}}{{f_{n}^{2}}}{b_{n}}} \vert } < \infty} \}$,
2. $(F_{p})^{\alpha}= \{ { ( {{b_{n}}} )\in\omega:\sup_{K \in\mathfrak{F} } \sum_{k} {{{ \vert {\sum_{n \in K\cap\{k,k+1\} } (-1)^{n-k}\frac{f_{k}f_{k+1}}{f_{n}^{2}}b_{n} } \vert }^{q}} < \infty} } \}$,
3. $(F_{\infty})^{\alpha}= \{ { ( {{b_{n}}} )\in\omega :\sup_{K \in\mathfrak{F} } \sum_{k} {{{ \vert {\sum_{n \in K\cap\{ k,k+1\}} (-1)^{n-k}\frac{f_{k}f_{k+1}}{f_{n}^{2}}b_{n} } \vert }} < \infty } } \}$,
4. ${ ( {{F_{1}}} )^{\beta}}={ ( {{F_{1}}} )^{\gamma}} = \{ { ( {{b_{n}}} ) \in\omega:\sup_{n,k \in \mathbb {N}} \vert {\sum_{n = k}^{k + 1} {{{( - 1)}^{n - k}}\frac {{{f_{k}}{f_{k + 1}}}}{{f_{n}^{2}}}{b_{n}}} } \vert < \infty} \}$,
5. ${ ( {{F_{p}}} )^{\beta}}={ ( {{F_{p}}} )^{\gamma}} = \{ { ( {{b_{n}}} ) \in\omega:\sup_{n \in\mathbb {N}} \sum_{k = 0}^{n} {{{ \vert {\sum_{j = k}^{k + 1} {{{( - 1)}^{j - k}}\frac{{{f_{k}}{f_{k + 1}}}}{{f_{j}^{2}}}{b_{j}}} } \vert }^{q}}} < \infty} \}$,
6. ${ ( {{F_{\infty}}} )^{\beta}} = \{ { ( {{b_{n}}} ) \in\omega:\sum_{k} { \vert {\sum_{j = k}^{k + 1} {{{( - 1)}^{j - k}}\frac{{{f_{k}}{f_{k + 1}}}}{{f_{j}^{2}}}{b_{j}}} } \vert }\textit{ converges uniformly in }n} \}$,
7. ${ ( {{F_{\infty}}} )^{\gamma}} = \{ { ( {{b_{n}}} ) \in\omega\,:\sup_{n \in{\Bbb {N}}} \sum_{k = 0}^{n} { \vert {\sum_{j = k}^{k + 1} {{{( - 1)}^{j - k}}\frac{{{f_{k}}{f_{k + 1}}}}{{f_{j}^{2}}}{b_{j}}} } \vert } < \infty} \}$.

Next consider the Euler sequence spaces of order *θ*, defined as

$$e_{p}^{\theta}= \left \{ {(x_{n} )\in\omega:\sum _{n = 0}^{\infty}\left \vert {\sum _{k = 0}^{n} \begin{pmatrix} n\\ k \end{pmatrix} } (1 - \theta)^{n - k} \theta^{k} x_{k} \right \vert ^{p}< \infty} \right \}\quad (1\le p < \infty) $$

and

$$e_{\infty}^{\theta}= \left \{ {(x_{n} )\in\omega:\sup _{n \in\mathbb{N}} { \left \vert {\sum_{k = 0}^{n} \begin{pmatrix} n\\ k \end{pmatrix} } (1 - \theta)^{n - k} \theta^{k} x_{k} \right \vert ^{p}< \infty} } \right \}, $$

which are the matrix domain of the Euler matrix in $\ell_{p}$ [1], where the Euler matrix $E(\theta)= (e_{n,k} )$ is defined by

$$e_{n,k} = \textstyle\begin{cases} \binom{n}{k}(1 - \theta)^{n - k} \theta^{k},&0 \le k \le n, \\ 0,& k > n. \end{cases} $$

Since the inverse of $E(\theta)$ is $E(\frac{1}{\theta})$, we observe that Theorems 4.4, 4.5, and 4.6 of [1] are all the special cases of Theorems 2.1, 2.2, and 2.3, respectively, in which the matrix *E* is replaced by $E(\theta)$.

We refer the readers to [1, 2, 4, 6], and [7] for some results which are all the special cases of Theorems 2.1, 2.2, and 2.3.

## Conclusions

In this study, we obtain the *α*-, *β*-, and *γ*-duals of the domain of an arbitrary invertible summability matrix *E* in $\ell_{p}$ and show that the recent works by Altay, Başar, and Mursaleen are all the special cases of our results.
